# Morning yoga as an embodied regulatory practice: longitudinal changes in children’s self-esteem and gratitude following participation in a school-based yoga program

**DOI:** 10.3389/fpsyg.2026.1884043

**Published:** 2026-07-23

**Authors:** Shoichi Shiota, Takahiro Ohitokata, Hiroshi Harada

**Affiliations:** 1 Faculty of Health Sciences, Department of General Education, Aomori University of Health and Welfare, Aomori, Japan; 2 Tahara Public Elementary School, Kitakyushu, Fukuoka, Japan; 3 Board of Education of Kitakyushu City, Kitakyushu, Fukuoka, Japan

**Keywords:** gratitude, school-based program, self-esteem, socio-emotional learning, yoga

## Abstract

**Background:**

Self-esteem and gratitude are important psychological constructs associated with children’s socio-emotional development and well-being. Previous studies have suggested that yoga may be associated with emotional regulation, attentional stability, and prosocial functioning. However, few studies have examined whether participation in school-based yoga programs is associated with changes in both self-esteem and gratitude among children. The present study aimed to examine longitudinal changes in self-esteem and gratitude following participation in a structured yoga program incorporating loving-kindness meditation in elementary school settings.

**Methods:**

This pilot study was conducted in seven public elementary schools in Kitakyushu City, Japan, as part of the regular school curriculum. A total of 401 children participated in a structured “Asa (morning) yoga” program conducted once weekly for 15 min from September 2025 to February 2026. The intervention included physical postures (asana), breathing techniques, attention-focused meditation, and loving-kindness meditation. Self-esteem was assessed using the Self-esteem Scale by Rosenberg for Elementary and Junior High School Students, and gratitude was assessed using the Interpersonal Gratitude Scale for Children. Pre–post changes were analyzed using paired *t*-tests.

**Results:**

Self-esteem scores were significantly higher at post-intervention than at pre-intervention. Gratitude scores were also significantly higher at post-intervention than at pre-intervention.

**Conclusion:**

Participation in a brief, structured school-based yoga program was associated with positive pre–post changes in self-esteem and gratitude over the study period. These findings suggest that participation in school-based yoga programs may be associated with positive changes in selected socio-emotional outcomes in children. However, given the absence of a control group, causal interpretations should be made with caution, and future controlled studies are needed to clarify the nature of these relationships.

## Introduction

Self-esteem and gratitude are among the most widely studied constructs in psychology. Self-esteem refers to an individual’s positive and negative evaluation of the self (Rosenberg, 1965). Gratitude, a positive emotion arising from recognizing and appreciating benefits received from others, has been linked to adolescents’ prosocial behavior and well-being (Froh et al., 2010; Ma et al., 2017). Both self-esteem and gratitude are shaped by significant others (e.g., parents, friends, and mentors) and are beneficial for children’s physical and psychological development (e.g., Chelvam and Ismail, 2020; Kerry et al., 2023; Orth and Robins, 2022), and their cultivation through education is considered important in Japan (Ministry of Education, Culture, Sports, Science and Technology, 2009). However, levels of self-esteem and gratitude among Japanese children have been reported to be lower than those in other countries (Cabinet Office, 2019). Yoga, an ancient mind–body practice, has been associated with positive psychological outcomes, including self-esteem and gratitude in children. The present study therefore aimed to examine longitudinal changes in self-esteem and gratitude among elementary school children following participation in a structured six-month school-based yoga program.

Self-esteem is an overall evaluation of an individual’s worth and ability and is strongly influenced by social feedback from parents, teachers, and peers (Du et al., 2017). Previous studies (e.g., Chelvam and Ismail, 2020; Guo et al., 2022; Hirano et al., 2025; Mertens et al., 2022; Swann et al., 2007) have consistently shown that high self-esteem is associated with better peer relationships, more satisfying romantic relationships, greater academic and occupational success, improved mental health, and higher psychological well-being. In children, Hosogi et al. (2007) reported that exposure to family-related adversities—such as parental psychiatric disorders, economic hardship, and child abuse—was associated with lower self-esteem and increased psychosomatic symptoms. Similarly, Yeung et al. (1999) and Wang et al. (2021) demonstrated that cohesion with parents and teachers is positively related to both children’s self-esteem and academic performance. In addition, Lavy and Naama-Ghanayim (2020) showed that teachers’ caring behaviors enhance both students’ self-esteem and well-being. Taken together, these findings suggest that self-esteem is fundamentally shaped through supportive and trusting interpersonal experiences. Therefore, approaches intended to support children with low self-esteem may benefit from enhancing positive interpersonal representations and experiences.

Gratitude is a generalized tendency to recognize and respond with grateful emotion to the benevolence of others in one’s positive experiences and outcomes (McCullough et al., 2002). Previous studies (e.g., Gilek, 2010; Komase et al., 2021) have demonstrated that gratitude is associated with higher self-esteem, subjective well-being, life satisfaction, and better mental health outcomes, including lower levels of depression and perceived stress. In addition, Chen and Zhu (2022) showed that gratitude reduces children’s bullying perpetration. Janik et al. (2025) further reported that gratitude enhances children’s well-being and academic outcomes. Taken together, these findings suggest that fostering both self-esteem and gratitude is essential for the healthy physical and psychological development of children. While previous studies have emphasized the role of interpersonal experiences in shaping these outcomes, individuals may differ in how they perceive and interpret such experiences, particularly in emotionally salient contexts (Szcześniak et al., 2022). The present framework assumes that such experiences influence self-related outcomes only in so far as they are symbolically represented and processed, with such processing fundamentally grounded in and constrained by bodily states.

Recent reviews have highlighted the important role of physical activity in supporting mental health among children and adolescents, although the strength of causal evidence varies across outcomes (Biddle et al., 2019). School-based yoga represents one form of movement-based practice that may be associated with children’s psychological well-being. Yoga is a long-standing physical, mental, and spiritual discipline with roots in India that aims to create a state of perfect balance and harmony (Black, 2023; Hoyez, 2007). The practice of yoga focuses on the integration of mind, body, and spirit through physical poses (asana), breathing exercises, and meditation techniques, including attention-focused meditation and loving-kindness meditation (Gothe et al., 2019). One’s awareness of both emotional reactions and bodily states may be enhanced during asana sequences with breathing techniques (Shiota and Nomura, 2018). Meditation combined with breathing techniques may promote cognitive reappraisal of psychologically salient experiences, thereby contributing to the development of cognitive schemas associated with equanimity and hopefulness. A growing body of research has demonstrated that yoga practice has been associated with a range of beneficial psychological outcomes. For example, Bhardwaj et al. (2017) and Janjhua et al. (2020) reported that yoga practice has been associated with improvements in children’s self-esteem. Similarly, Reppa (2017) and Sahu (2023) demonstrated that yoga programs have been associated with improvements in prosocial behavior in children. Loving-kindness is a concept originating from Buddhism that emphasizes moving beyond self-interest toward compassion, care, and love for others and the broader world (Hofmann et al., 2015; Shiota and Nomura, 2018). Loving-kindness meditation (LKM), which is often included as a component of yoga practice, traditionally involves cultivating compassion and forgiveness toward all beings, including oneself (Halverson and Petersen, 2024). Previous studies (e.g., Iwamoto et al., 2020; Malin and Gumpel, 2022) have shown that LKM has been associated with increased altruistic behavior. In addition, Wang et al. (2021) found that LKM has been associated with improvements in self-evaluation, self-acceptance, and social relationships. Based on this body of evidence, and consistent with the observation by Szcześniak et al. (2022), yoga incorporating LKM may enhance both self-related and other-related outcomes, such as self-esteem and gratitude. However, to the best of our knowledge, relatively few studies have directly examined these combined effects. Yoga practice may be associated with reductions in emotional reactivity, which may in turn influence mental representations of both the self and others. We hypothesized that yoga practice incorporating loving-kindness meditation would be associated with reduced emotional reactivity and corresponding changes in mental representations of the self and others, as reflected in self-related (e.g., self-esteem) and other-related outcomes (e.g., gratitude and prosocial behavior). The present study aimed to examine changes in self-esteem and gratitude following participation in a structured yoga program. Breathing techniques, attention-focused meditation, and loving-kindness meditation were operationalized based on previously described definitions (Shiota, 2024; Shiota and Nomura, 2018). The yoga postures and movement-based exercises (asanas) were adapted from established child-friendly practices and standardized for implementation in school-based settings, as detailed in the Supplementary materials. Self-esteem and gratitude were selected as primary outcomes because they are commonly used indicators in school-based socio-emotional intervention research and are considered sensitive to changes in children’s educational and psychosocial environments. Both constructs have been associated with children’s psychosocial adjustment, well-being, and positive interpersonal functioning (de Carvalho et al., 2017; Hall et al., 2025; Iwahori et al., 2022).

## Method

### Participants and intervention procedures

Before starting this intervention study, ethical approval for the study was obtained from the Kitakyushu City Board of Education and the Committee of Physical Education Research of Kitakyushu City prior to implementation. The study was conducted as a school-based educational activity within participating elementary schools under the supervision of the Kitakyushu City Board of Education and the relevant educational authorities. Approval for participation was also obtained from the principals of the participating schools. The study procedures were explained to parents and guardians through school meetings conducted by the principals. Participation in questionnaire assessments and the use of data for research purposes were voluntary. All data were anonymized prior to analysis, and participant confidentiality and privacy were protected throughout the study. The school-based yoga program was implemented as part of routine school activities and was offered to all participating children, including those enrolled in special education classrooms. However, children enrolled in special education classrooms for intellectual or emotional disabilities were excluded from the present analyses because their educational programs differed substantially from those of general education classrooms and the study measures were not specifically validated for these populations. Accordingly, the findings of the present study are limited to children in general education classrooms and should not be generalized to children receiving special education services. For the present analyses, only participants with both pre- and post-intervention questionnaire data and valid responses on the outcome measures were included. No additional exclusions were applied, and all eligible participants with complete data were analyzed. Because the intervention was implemented as part of routine school-based activities, no formal attrition analysis was conducted. The “Asa (morning) yoga” program was conducted once weekly during Monday morning homeroom sessions from September 2025 to February 2026. Each session lasted approximately 15 min. All children attending the seven participating public elementary schools took part in the program as part of regular school activities. To minimize variability in the intervention procedures, all yoga sessions were conducted by a single male practitioner (the first author, SS) with 7 years of yoga experience and 8 years of clinical experience. Data from 401 children (170 boys and 231 girls; *mean* age = 10.56 years, *SD* = 1.16) for whom informed consent was obtained were included in the analyses. The primary outcome measures were self-esteem and gratitude, assessed before and after the intervention period.

### Asa (morning) yoga sequence

The yoga sequence was developed collaboratively by the first author and the second author, an experienced elementary school principal and physical education specialist. The selected postures were intended to promote core muscle engagement, balance, flexibility, and bodily awareness, while the meditation components were included to encourage calm attention and support children’s adjustment to daily school life. Prior pilot implementation indicated that the sequence was feasible and safe for elementary school children, with no injuries or adverse events reported. Consistent with these preliminary observations, no adverse events or injuries were reported during the present study. The Asa (morning) yoga program consisted of a structured sequence of physical postures (asana), breathing techniques, attention-focused meditation, and loving-kindness meditation. The asana sequence included Urdhva Hastasana (Upward Salute), Vrksasana (Tree Pose), Beetle Pose, Stag Beetle Pose, and Traditional sumo-inspired stomping and controlled leg-raising movements. Following the asana sequence, participants engaged first in attention-focused meditation and subsequently in loving-kindness meditation. The intervention protocol was delivered in a fixed and standardized order across all sessions and schools to ensure consistency of implementation. The program was designed for feasibility within school settings and targeted basic aspects of children’s motor and attentional functioning, including balance, core engagement, attentional stability, and bodily awareness. A detailed description of the intervention protocol is provided in the Supplementary materials.

### Measures

#### Self-esteem scale by Rosenberg for Elementary and Junior High School Students

Self-esteem was assessed using the Self-esteem Scale by Rosenberg for Elementary and Junior High School Students (Suzaki and Anii, 2013). This scale consists of eight items assessing children’s self-esteem. Responses are rated on a 5-point Likert scale ranging from 1 (“Does not describe me well”) to 5 (“Describes me very well”). Higher scores indicate higher levels of self-esteem.

#### Interpersonal Gratitude Scale for Children

Gratitude was assessed using the Interpersonal Gratitude Scale for Children (Fujiwara et al., 2014). This scale consists of eight items evaluating children’s gratitude toward others. Responses are rated on a 5-point Likert scale ranging from 1 (“Does not describe me well”) to 5 (“Describes me very well”). Higher scores indicate higher levels of gratitude.

Internal consistency was acceptable for all measures. Cronbach’s alpha coefficients for the Self-esteem Scale by Rosenberg for Elementary and Junior High School Students were 0.74 at pre-intervention and 0.77 at post-intervention. For the Interpersonal Gratitude Scale for Children, Cronbach’s alpha coefficients were 0.91 at pre-intervention and 0.92 at post-intervention.

### Statistical analysis

For self-esteem, skewness and kurtosis values were 0.14 and −0.22 at pre-intervention and 0.18 and −0.31 at post-intervention, respectively. For interpersonal gratitude, skewness and kurtosis values were −0.54 and −0.77 at pre-intervention and −0.85 and −0.34 at post-intervention, respectively. All skewness and kurtosis values were within commonly accepted ranges (|skewness| < 1, |kurtosis| < 1), indicating no substantial deviations from normality, supporting the use of parametric analyses.

Paired-samples *t*-tests were conducted to examine changes in self-esteem and gratitude from pre-intervention to post-intervention. Effect sizes were calculated using Cohen’s *d* values, and 95% confidence intervals were reported. In addition, to examine potential school-level effects, mixed-design repeated-measures analyses of variance (ANOVAs) were conducted with time (pre- vs. post-intervention) as a within-subject factor and school as a between-subject factor. These analyses were performed to assess whether observed changes differed across the participating schools.

## Results

### Paired *t*-test analyses of pre–post changes in self-esteem and gratitude

**Figure 1:**
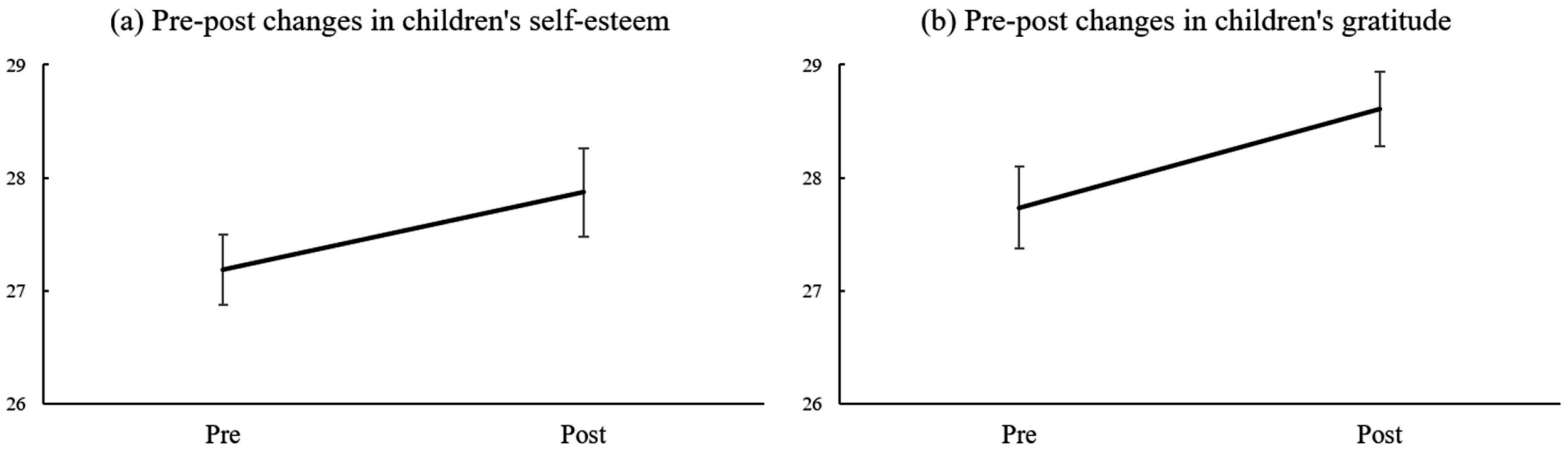
Mean self-esteem and interpersonal gratitude scores before and after the school-based yoga program. Error bars indicate 95% confidence intervals. **(a)** Self-esteem scores. **(b)** Interpersonal gratitude scores among elementary school children.

Self-esteem scores were significantly higher at post-intervention than at pre-intervention (*t*(400) = 2.82, *p* = 0.005, *d* = 0.14, 95% CI [0.04, 0.24]). Gratitude scores were also significantly higher at post-intervention than at pre-intervention (*t*(400) = 4.42, *p* < 0.001, *d* = 0.22, 95% CI [0.12, 0.32]) (Figure 1).

### Repeated-measures ANOVA examining changes in self-esteem and gratitude across schools

To examine potential clustering effects at the school level, a mixed-design repeated-measures ANOVA was conducted with time (pre- vs. post-intervention) as a within-subject factor and school as a between-subject factor. For self-esteem scores, the analysis revealed a significant main effect of time [*F*(1, 394) = 7.54, *p* = 0.006, *η_p_*^2^ = 0.02]. The main effect of school was not statistically significant [*F*(6, 394) = 0.60, *p* = 0.73]. In addition, the time × school interaction was not significant [*F*(6, 394) = 1.48, *p* = 0.19], indicating that the observed pre–post changes in self-esteem did not significantly differ across schools. For interpersonal gratitude, the analysis revealed a significant main effect of time [*F*(1, 394) = 20.51, *p* < 0.001, *η_p_*^2^ = 0.05]. Neither the main effect of school [*F*(6, 394) = 1.70, *p* = 0.10] nor the time × school interaction [*F*(6, 394) = 0.98, *p* = 0.44] was statistically significant, indicating that the observed pre–post changes in gratitude did not significantly differ across schools (Table 1).

**Table 1:** Pre-intervention and post-intervention scores for self-esteem and interpersonal gratitude across participating schools.

Measure	School	Pre	Post	*t*-value	Cohen’s *d*	95% CI
SSREJ	School A	26.85 (3.65)	27.41 (3.94)			
	School B	28.17 (4.49)	27.00 (4.21)			
	School C	26.25 (3.91)	28.55 (4.08)			
	School D	27.56 (3.69)	27.97 (3.68)			
	School E	27.49 (3.89)	28.14 (4.12)			
	School F	26.90 (4.00)	27.84 (3.87)			
	School G	25.85 (3.55)	27.85 (4.66)			
	Total	27.19 (3.19)	27.87 (3.99)	2.82	0.14	0.04, 0.24
IGSC	School A	27.34 (3.88)	29.15 (2.93)			
	School B	27.62 (3.85)	28.28 (3.69)			
	School C	25.80 (3.87)	27.80 (3.22)			
	School D	28.20 (3.39)	28.64 (3.64)			
	School E	27.45 (3.81)	28.49 (3.33)			
	School F	28.33 (3.29)	28.95 (3.17)			
	School G	26.93 (3.50)	27.85 (3.62)			
	Total	27.74 (3.64)	28.61 (3.40)	4.42	0.22	0.12, 0.32

Values are presented as mean (SD). SSREJ = Self-esteem Scale by Rosenberg for. Elementary and Junior High School Students; IGSC = Interpersonal Gratitude Scale for Children.

## Discussion

In the present study, we examined changes in children’s self-esteem and gratitude following participation in a structured yoga program. The results indicated increases in both constructs across the study period. The present findings are broadly consistent with previous reports of positive psychological and socio-emotional changes following school-based yoga programs among children and adolescents (e.g., Sumner et al., 2025; Velásquez et al., 2015). While many previous studies have focused on secondary school or mixed-age populations, evidence from elementary school settings remains comparatively limited (e.g., Sivashankar et al., 2022). Therefore, the present findings add to the existing literature by providing additional evidence regarding longitudinal pre–post associations following participation in school-based yoga programs among elementary school children. Expanding the evidence base across diverse child populations is particularly important given growing evidence that appropriately adapted movement-based interventions may support mental health and well-being among children and adolescents with disabilities (Lee et al., 2025). Although statistically significant increases in self-esteem and gratitude were observed, the effect sizes were small. This suggests that the magnitude of change at the individual level was modest and should be interpreted cautiously. However, the intervention was brief, low-cost, and implemented within routine school activities. From a population-level perspective, even modest pre–post changes may have practical relevance if consistently observed across large numbers of children in educational settings. Nevertheless, further research is needed to determine whether these changes translate into meaningful educational, behavioral, or psychological outcomes over time.

Self-esteem is shaped through interpersonal and emotional experiences (Ghosh, 2024; Kramer et al., 2023). Previous studies have suggested that yoga may reduce emotional reactivity (Daly et al., 2015; Mocanu et al., 2018; Madhava Chandran et al., 2023). For example, Mocanu et al. (2018) reported that yoga practice decreased the intensity of self-reported emotional experiences, while Daly et al. (2015) showed that yoga interventions significantly improved emotional regulation. According to Shiota (2024), the cervical spine, thoracic spine, lumbar spine, and pelvis are actively engaged during asana sequences, which has been proposed to potentially contribute to functional changes in the autonomic nervous system and hormones such as oxytocin, which are associated with emotional reactivity. These findings suggest that physical and emotional regulation through yoga may be associated with processes that influence the way children process self-evaluative experiences. However, these mechanisms were not directly assessed in the present study. Gratitude is closely associated with social–emotional openness and positive interpersonal connectedness (Basit et al., 2024; Bono and Sender, 2018). Previous studies have suggested that emotional regulation and mindful attention facilitate openness to positive interpersonal experiences and social connectedness (Bayram et al., 2025; Lemay et al., 2025; Meneghini et al., 2024; Quaglia et al., 2015; Lopes et al., 2005; Wilson et al., 2020). Yoga may be associated with these processes by reducing emotional reactivity and promoting physiological regulation (Daly et al., 2015; Mocanu et al., 2018; Madhava Chandran et al., 2023; Shiota, 2024). However, the present study did not directly measure these processes, and their potential role should therefore be considered speculative.

To the extent that children become emotionally and physically more stable, they may become more open and receptive to positive interpersonal experiences, which could enhance feelings of gratitude. In addition, yoga is often practiced in a calm and supportive environment, which may further encourage social connectedness and interpersonal awareness. Our structured yoga sequences may be associated with changes in children’s bodily regulation, which could potentially be associated with reduced emotional hyperreactivity and increased attentional stability. These changes, if present, may, in turn, influence self- and other-related representations and promote more adaptive socio-emotional processing. However, these proposed pathways remain theoretical interpretations because no direct measures of emotional regulation, emotional reactivity, mindfulness, or related mediating variables were included in the present study. The structured yoga sequence used in this study can be implemented once a week in a short session. The intervention is highly standardized, easy to deliver to diverse populations, and no adverse events or injuries were reported. It may therefore be feasible to integrate this program into school-based educational settings. Accordingly, this structured yoga sequence may represent a potentially useful complement to socio-emotional learning and could be considered as a complementary approach to conventional psychological education. However, given the absence of a control group, these interpretations should be considered preliminary and require confirmation in future controlled studies.

Several limitations should be acknowledged in the present study. First, the absence of a control group limits causal interpretation of the findings. Pre–post changes observed over the study period may reflect factors other than the yoga program itself, including maturation, seasonal variation, repeated measurement effects, school experiences, or other concurrent influences. Therefore, the present findings should be interpreted as preliminary evidence of associations observed over time rather than definitive evidence of intervention effectiveness. However, the absence of a control group reflects practical constraints in Japanese school settings, where implementing randomized controlled designs is often difficult due to ethical and operational considerations within regular educational curricula. Second, the study relied solely on self-report measures, which may be subject to response bias. Third, despite the multi-school design, the potential influence of teacher- and school-level effects could not be fully controlled, which may have affected the observed outcomes. Fourth, the observed effect sizes were small, indicating that the magnitude of the detected changes was modest. Although statistically significant differences were identified, the practical significance of these changes for children’s everyday psychological functioning remains unclear. Future studies should examine whether the observed pre–post changes are sustained over time and whether they are associated with meaningful educational, behavioral, or mental health outcomes. Fifth, the present study did not include direct measures of emotional regulation, emotional reactivity, mindfulness, or related psychological processes. Consequently, the mechanisms through which participation in the yoga program may have been associated with changes in self-esteem and gratitude remain speculative and should be investigated in future studies. Sixth, while the yoga program was implemented as part of routine school activities and was available to all participating children, the present analyses included only children enrolled in general education classrooms. Children receiving special education services were excluded from the analyses because the outcome measures were not specifically validated for these populations and their educational programs differed substantially. Therefore, the present findings should not be interpreted as evidence that the observed pre–post associations generalize to all children. Future studies should also examine the feasibility, accessibility, and effectiveness of implementing similar school-based yoga or movement programs among more diverse groups of children, including those with special educational needs. Such research would help determine whether these programs can be adapted to meet the needs of a broader range of learners while maintaining their practicality within routine school settings. Finally, the generalizability of the findings is limited to similar school-based contexts and populations. Although additional analyses indicated that the observed changes did not significantly differ across participating schools, the present study did not assess teacher-level implementation differences or other classroom-specific contextual factors. Variations in teacher engagement, classroom climate, and other unmeasured environmental influences may have contributed to the observed outcomes. Future studies should examine these factors more explicitly to better understand the conditions under which school-based yoga programs may be associated with positive socio-emotional outcomes. The present study did not formally assess intervention fidelity or participants’ adherence to the yoga sessions. Although the program was implemented as part of routine school activities, variations in how the intervention was delivered across classrooms or the extent of individual participation may have influenced the observed outcomes. Future studies should include systematic assessments of implementation fidelity and exposure to better understand dose–response relationships in school-based yoga programs.

## Data Availability

The raw data supporting the conclusions of this article will be made available by the authors, without undue reservation.
