# Supplementary material for: Morning yoga as an embodied regulatory practice: longitudinal changes in children’s self-esteem and gratitude following participation in a school-based yoga program
Source: Front Psychol. 2026 Jul 23;17:1884043. doi: 10.3389/fpsyg.2026.1884043 (PMC13441743; doi:10.3389/fpsyg.2026.1884043)
Supplement: Supplementary file 1 [file Supplementary_file_1.DOCX]

**Structured Yoga Sequence: Implementation Procedure**

The structured yoga sequence was implemented as a 15-minute group-based session conducted once per week within regular school activities. Each session followed a standardized and fixed order to ensure consistency across schools and instructors.

At the beginning of each session, children performed a sequence of yoga postures and movement-based activities (approximately 5 minutes), including standing, balancing, and stretching exercises. Each posture was demonstrated by the instructor and performed by the class under verbal guidance. The structured sequence was performed in the following order: Urdhva Hastasana (Upward Salute), Vrksasana (Tree Pose), Beetle Pose or Stag Beetle Pose (alternated weekly), traditional sumo-inspired stomping and controlled leg-raising movements, and finally Urdhva Hastasana (Upward Salute).

**〇 Urdhva Hastasana (Upward Salute)**

A standing posture involving full arm extension overhead with gentle stretching of the upper body. While maintaining the posture, children performed three cycles of diaphragmatic breathing. This posture was used to promote spinal extension, postural alignment, and body awareness.

**〇 Vrksasana (Tree Pose)**

A single-leg balance posture in which one foot is placed on the opposite leg while maintaining an upright posture. The supporting leg was alternated between the left and right sides. While maintaining the posture, children performed three cycles of diaphragmatic breathing. This posture was used to support balance control, concentration, and postural stability.

**〇 Beetle Pose**

A standing posture performed with the feet placed at approximately double shoulder-width apart, with the toes directed outward. The hands were brought together in front of the body to form a bowl-like shape. This was followed by five repetitions of a controlled squat movement. This exercise was used to promote lower-limb strength, coordination, and body awareness.

**〇 Stag Beetle Pose**

A standing posture performed with the feet placed at approximately double shoulder-width apart, with the toes directed outward. Both arms were extended upward toward the ceiling with a slight flexion at the elbows. This was followed by five repetitions of a controlled squat movement. This exercise was used to promote lower-limb strength, coordination, and postural control.

**〇 Traditional sumo-inspired stomping and controlled leg-raising movements**

A standing posture performed with the feet placed at approximately double shoulder-width apart, with the toes directed outward. Body weight was shifted onto the right leg, and the left heel was brought toward the right heel before being lifted upward. Participants maintained a single-leg stance on the right leg for approximately 3 seconds. The left foot was then slowly returned to the ground, followed by a transition into a semi-squat position. The movement was then repeated on the opposite side, for a total of 10 repetitions (5 per side). This exercise was used to promote balance, lower-limb strength, and postural control.

Next, children engaged in attention-focused meditation (approximately 6 minutes), during which they were instructed to observe their breathing and bodily sensations while gently returning attention when distractions occurred.

**〇 Attention-Focused Meditation (Guided Instruction)**

Participants were instructed to close their eyes and place their hands on their knees or in a comfortable position. They were then guided to gently sway their upper body in a pendulum-like motion from side to side. The movement was gradually enlarged to enhance awareness of pelvic and lower-body sensations. Once awareness of pelvic movement was established, participants gradually reduced the amplitude of the movement and returned to a stable, centered position based on their own bodily sensations.

Following this, participants performed paced breathing under instructor guidance. Each cycle consisted of inhalation through the nose followed by exhalation through the nose. A total of 15 cycles were performed. For the first 10 cycles, inhalation was guided for approximately 5 seconds and exhalation for approximately 6 seconds. For the final 5 cycles, inhalation was extended to approximately 6 seconds and exhalation to approximately 8 seconds.

Finally, the session concluded with loving-kindness meditation (approximately 4 minutes), in which children were guided to silently repeat simple phrases directed toward themselves and others, with the aim of fostering positive emotional states and interpersonal awareness.

**〇 Loving-kindness meditation (Guided Instruction)**

As a continuation of the attention-focused meditation, participants were instructed to keep their eyes closed and to continue breathing in a natural and comfortable rhythm at their own pace.

Participants were then guided to recall as many positive experiences as possible involving a specified target group (family members, teachers, or friends; the target group was changed weekly) over the past year. They were instructed that multiple individuals could be included in their recollections. This recall phase was conducted for 2 minutes.

After the recall period, participants were instructed to silently express feelings of gratitude toward the individuals they had recalled. This gratitude expression phase was also conducted for 2 minutes.

All sessions were conducted in regular classroom settings. In Japan, classrooms accommodating approximately 30 children allowed sufficient space for practice by arranging desks and chairs toward the back of the room, creating an open area of approximately 1 m × 1 m per child. The meditation components were performed in a seated position on chairs.

All sessions were delivered by trained school staff following a standardized protocol developed by the authors. The sequence, duration, and instructional procedures were consistent across all participating schools. No adverse events or injuries were reported during the intervention period. Given that the intervention was designed for children, the meditation components were intentionally simplified to ensure feasibility, clarity of instruction, and age-appropriate implementation within school settings.
